# Effects of Spaceflight and Simulated Microgravity on YAP1 Expression in Cardiovascular Progenitors: Implications for Cell-Based Repair

**DOI:** 10.3390/ijms20112742

**Published:** 2019-06-04

**Authors:** Victor Camberos, Jonathan Baio, Leonard Bailey, Nahidh Hasaniya, Larry V. Lopez, Mary Kearns-Jonker

**Affiliations:** 1 Department of Pathology and Human Anatomy, Loma Linda University School of Medicine, Loma Linda, CA 92350, USA; vcamberos@llu.edu (V.C.); jonbaio13@gmail.com (J.B.); lvlopez@llu.edu (L.V.L.); 2 Department of Cardiovascular and Thoracic Surgery, Loma Linda University School of Medicine, Loma Linda, CA 92350, USA; llbailey@llu.edu (L.B.); nhasaniya@yahoo.com (N.H.)

**Keywords:** microgravity, spaceflight, YAP1, cardiac

## Abstract

Spaceflight alters many processes of the human body including cardiac function and cardiac progenitor cell behavior. The mechanism behind these changes remains largely unknown; however, simulated microgravity devices are making it easier for researchers to study the effects of microgravity. To study the changes that take place in cardiac progenitor cells in microgravity environments, adult cardiac progenitor cells were cultured aboard the International Space Station (ISS) as well as on a clinostat and examined for changes in Hippo signaling, a pathway known to regulate cardiac development. Cells cultured under microgravity conditions, spaceflight-induced or simulated, displayed upregulation of downstream genes involved in the Hippo pathway such as YAP1 and SOD2. YAP1 is known to play a role in cardiac regeneration which led us to investigate YAP1 expression in a sheep model of cardiovascular repair. Additionally, to mimic the effects of microgravity, drug treatment was used to induce Hippo related genes as well as a regulator of the Hippo pathway, miRNA-302a. These studies provide insight into the changes that occur in space and how the effects of these changes relate to cardiac regeneration studies.

## 1. Introduction

Space exploration is expanding rapidly and broadening our understanding of the world around us, as well as the cells within us. Technological advancements now allow us to perform cell culture studies aboard the International Space Station (ISS) to identify the molecular changes that take place in an environment of microgravity [1,2,3,4].

Several cell types have been cultured in space such as bone marrow progenitor cells [2], mouse embryonic stem cells [5], endothelial cells [3], and human cardiac progenitor cells (CPCs) [4]. Microgravity has been shown to have both beneficial and detrimental effects on various cell types. For example, culture in microgravity conditions has been shown to cause hypertrophy in osteoblast cells [6] and inhibits differentiation in mouse embryonic stem cells [5], but beneficial effects include an increase in the expression of transcripts encoding DNA repair genes in human fibroblasts [7] and an increase in migratory capacity in CPCs [4]. Upon reloading in normal gravity conditions, microgravity exposure during spaceflight can be beneficial. For example, Blaber et al. provided evidence that bone marrow derived mesenchymal progenitor cells harvested from mice flown in space exhibit greater osteogenesis potential in vitro under normal gravitational force [8]. This study emphasizes the finding that transcriptional alterations in space can influence cell responses upon return to normal gravity and that the duration of exposure plays a significant role in the biological impact of microgravity. Studies performed on the ISS have begun to provide insight into these processes but opportunities for spaceflight culture are limited and take years to plan and complete. Alternative methods of studying microgravity play an important role in understanding the impact of microgravity on cell biology and how this information may be applied on Earth as well as in space.

Simulated microgravity studies are currently being performed by several laboratories, and in some cases, the opportunity to directly compare simulated microgravity findings with similar studies performed on the ISS has provided insight regarding where similarities and differences may be expected [9,10]. Simulated microgravity is achieved through the use of clinostats, which are devices that rotate cells continuously around one axis, 2D, perpendicular to the force of gravity, or two axes, 3D, to achieve weightlessness similar to the weightlessness experienced in space [11,12]. Samples being studied in clinostats are easily accessible and can be maintained on a regular schedule. The use of such devices has shown that simulated microgravity as a model can achieve transcriptional changes and/or functional effects in cells that are similar to those reported after culture on the ISS [4,13]. Yuge et al. has demonstrated the benefits of simulated microgravity specifically in terms of proliferation and survival [14]. Additionally, this group has shown that mouse bone marrow stromal cells used for transplant following simulated microgravity exposure showed higher proliferation, survival, and regenerative capacity for spinal cord injury [15,16]. These findings further emphasize the possibility that microgravity, whether simulated or spaceflight-induced, may prove to be beneficial for other regenerative and transplant studies; however, the mechanism is not well understood. Further comparative studies are needed to validate simulated microgravity as an appropriate model of spaceflight.

Research in our own lab has shown that CPCs cultured on the ISS or on a clinostat are more proliferative and demonstrate elevated levels of transcripts encoding growth factors and DNA repair genes [4,13,17]. One of the most significant transcripts impacted by microgravity exposure of adult CPCs cultured on the ISS was yes-associated protein (YAP1), a key component of the Hippo signaling pathway, which regulates cell proliferation and is responsible for controlling organ growth [18,19,20,21]. YAP1 expression is well-documented as relevant for promoting cell survival and inhibiting apoptosis as well as promoting repair in regenerative studies [22,23]. The influence of simulated microgravity on YAP1 expression in adult CPCs, if determined, may therefore provide information relevant for cell therapy on Earth. The application of this information to stem cell-based treatment for heart disease has the potential to significantly improve the limited efficacy of current stem cell-based therapies. Adults, unlike neonates, are unable to functionally restore lost tissue following injury [24,25,26]. Thus, the possibility of conditioning adult CPCs with short term exposure to microgravity to overexpress transcripts, including YAP1, which promote the survival of cardiac stem cells capable of repair could unexpectedly be beneficial. This novel path may be worth pursuing as a potential pretreatment and therapy for myocardial infarction patients on Earth, as high levels of YAP1 protect cells during differentiation and aid in improving cardiac function [27,28,29]. The following study is focused on the impact of microgravity experienced in spaceflight and simulated microgravity on the Hippo-YAP signaling pathway in cardiovascular progenitor cells. Additionally, we present potential applications to cardiovascular stem cell transplantation in a clinically relevant model of cardiovascular repair.

## 2. Results

### 2.1. Human Islet-1+ Cardiac Progenitor Cells co-Express Several Markers of Early Cardiovascular Differentiation

**Figure 1:**
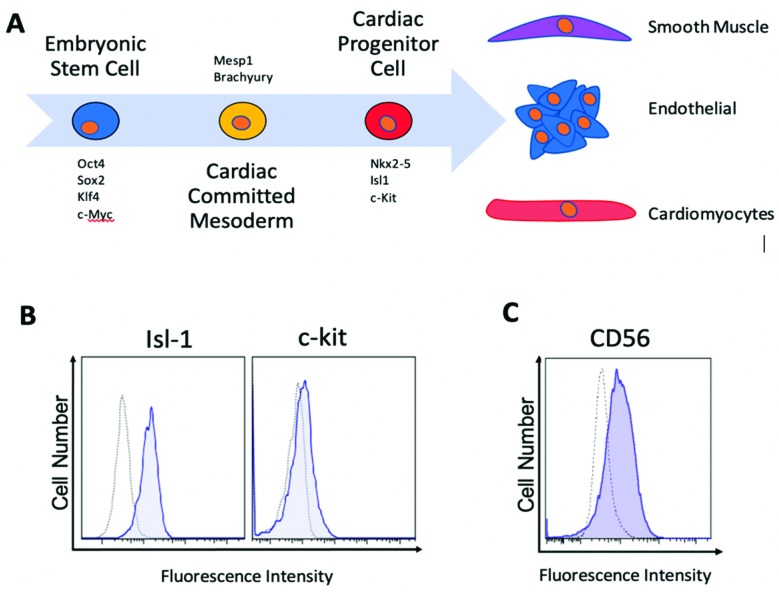
Patient-derived human cardiac progenitor cells (CPCs) can be isolated and selected for expansion as clones that express markers of early stage progenitors. A schematic representation of the cardiovascular differentiation pathway is shown in (**A**). These cells are isolated based on their co-expression of high levels of islet-1 and low levels of c-kit (**B**). Cells at this early stage express the glycoprotein CD56 (**C**).

The early cardiovascular progenitor cells (CPCs) cultured on the ISS and on the clinostat in our laboratory were derived from discarded cardiovascular tissue from human patients who have undergone clinically necessary procedures. Cell clones were isolated by single cell expansion and screened for the expression of markers specific to early cardiac progenitor cells as previously reported by our laboratory [30]. In addition, they are mesoderm-committed and express markers indicative of early stage progenitors, including SSEA1, Mesp1, and PDGFRα. These patient-derived CPCs are unique based on their early stage marker profile at isolation (Figure 1A), clonal derivation and selection by co-expression of high levels of islet-1 and low levels of c-Kit (Figure 1B). Islet-1 has been shown to be a definitive marker of a cardiac progenitor cell [31,32,33]. These cells also express CD56 (Figure 1C), which has been recently identified on the earliest stage of mesoderm-committed progenitor cells [34] and are capable of differentiating into the three lineages of the heart [35].

### 2.2. Hippo Signaling Pathway Activity Differs in Adult and Neonatal Cardiac Progenitor Cells

**Figure 2:**
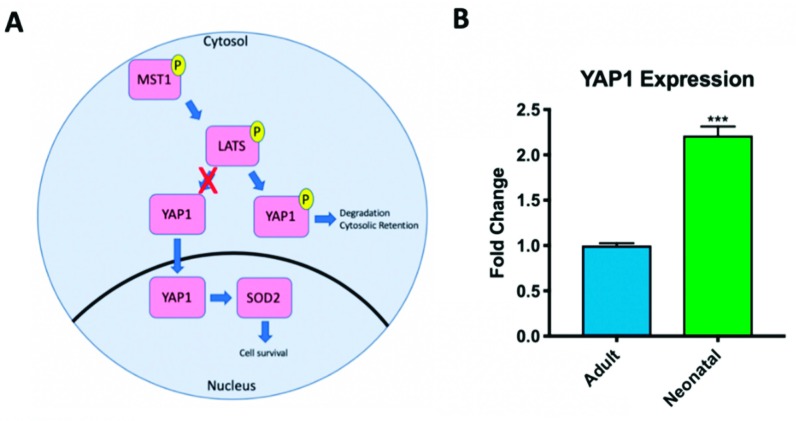
The Hippo signaling pathway phosphorylates and inactivates YAP1 when the pathway is active (**A**). Adults have limited expression of active YAP1 compared to neonates (**B**). *** *p* < 0.001. Fold changes are shown as the mean ± SEM. All samples were run in triplicate.

The Hippo signaling pathway was first discovered in *Drosophila* and later identified in mammals [36]. When active, this pathway consists of a kinase cascade in which YAP1 is directly phosphorylated, and inactivated, by large tumor suppressor kinase 1 and 2 (LATS1&2) (Figure 2A). A key characteristic of phosphorylated/inactive YAP1 is cytosolic retention and apoptosis which prevents the expression of downstream targets involved in cell survival and proliferation [37]. When the Hippo pathway is inhibited, active YAP1 is free to translocate into the nucleus and downstream targets such as SOD2, a marker of cell survival, are expressed. SOD2 alleviates the negative effects of reactive oxygen species released by cell stress or apoptosis [38,39].

Using RT-PCR, we measured baseline expression levels of YAP1 in neonatal (8d–1-month-old) and adult (57–72-year-old) human CPCs isolated in our laboratory. Consistent with results published by Gise et al. which showed that YAP1 transcripts are abundant in the neonatal mouse heart but decline with age [37], we show that YAP1 transcript levels in neonatal human CPCs are significantly higher than YAP1 levels in adult CPC clones isolated on the basis of comparable markers (Figure 2B) (2.214 ± 0.171-fold change, *p* < 0.001). Additionally, neonatal CPCs have been shown to be more proliferative than mature adult CPCs [30].

### 2.3. Microgravity Conditions Increase YAP1 and SOD2 Expression in Adult CPCs

**Figure 3:**
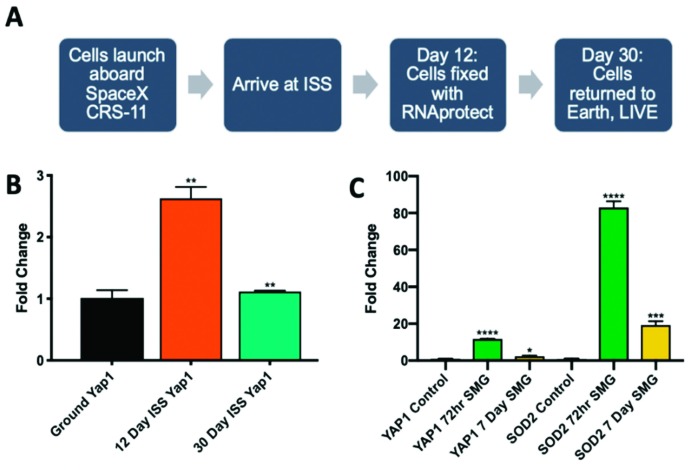
Human cardiac progenitor cells were launched into space and cultured aboard the International Space Station (ISS). Cells were fixed at 12 days or allowed to grow for 30 days before being returned to earth and fixed in RNAprotect (**A**). The expression of YAP1 in adult CPCs cultured aboard the ISS was increased after the 12-day time point and declined at 30 days (**B**). Similarly, adult CPCs cultured in simulated microgravity conditions expressed higher levels of YAP1, as well as the downstream target SOD2 (**C**) (*n* = 4 at 72 h, *n* = 3 at 7 days, representative sample shown in the figure). * *p* < 0.05, ** *p* < 0.01, *** *p* < 0.001, **** *p* < 0.0001. Fold changes are shown as the mean ± SEM. All samples were run in triplicate.

YAP1 expression in adult CPCs was observed in two different microgravity settings. First, adult CPCs were cultured aboard the ISS to measure the molecular changes that occur in a real microgravity environment (Figure 3A). After 12 days aboard the ISS, adult CPCs expressed higher YAP1 levels by nearly threefold compared to ground controls before regressing back to normal expression by day 30 (Figure 3B): 12 day (2.629 ± 0.186-fold change, *p* < 0.01), 30 day compared to 12 day (−1.512 ± 0.014-fold difference, *p* < 0.01). Next, adult CPCs were observed under simulated microgravity conditions via 2D clinorotation. After 72 h, cell number increased by 1.7-fold and both YAP1 as well as its downstream target SOD2, were significantly upregulated compared to cells cultured in comparable hardware under normal gravity conditions (Figure 3C): YAP1 after 72-h of simulated microgravity (11.76 ± 0.114-fold change, *p* < 0.0001), SOD2 after 72-h of simulated microgravity (83.05 ± 3.34-fold change, *p* < 0.0001). After 7 days of clinorotation, cell number increased 3.8-fold; however, YAP1 and SOD2 transcripts were lower compared to the 72h group: YAP1 after 7 days of simulated microgravity (2.364 ± 0.334-fold change, *p* < 0.05), SOD2 after 7 days of simulated microgravity (19.28 ± 2.17-fold change, *p* < 0.001). The upregulation of YAP1 in simulated microgravity follows the same trend as microgravity experienced in spaceflight in that there is an initial increase of expression followed by a decline. SOD2 expression mimics the trend of YAP1 with more dramatic fold changes.

### 2.4. YAP1 Expression is Elevated in the Cardiovascular Repair Zone When YAP1 Expressing Neonatal CPC Are Introduced Following Myocardial Infarction

**Figure 4:**
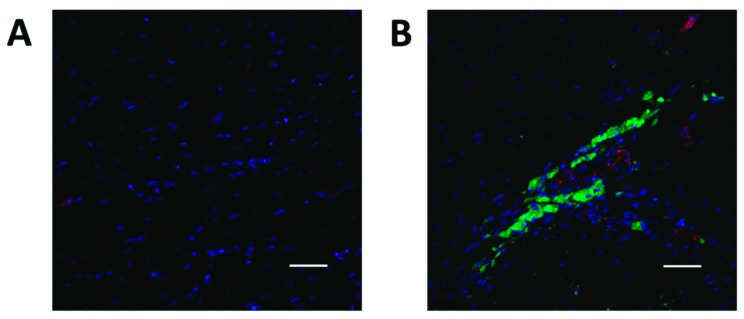
Neonatal sheep cardiovascular progenitor cell retention in the post-infarct repair region. In the non-infarcted region of the left ventricle, newly introduced cells were not identified (**A**). Carboxyfluorescein succinimidyl ester (CFSE) labeled (green) cells were identifiable in the region surrounding the infarction (**B**). Anti-von Willebrand Factor labeled endothelial cells were identified using Alexa Fluor 647 (red). Scale bar: 50 μm.

**Figure 5:**
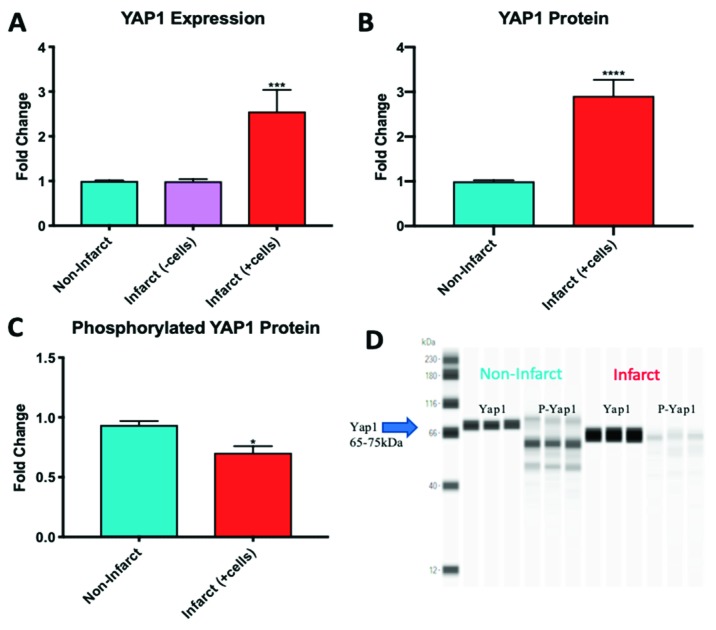
Infarct zones of sheep treated with cells express higher levels of active YAP1 when compared with non-infarcted regions as shown by PCR (**A**). YAP1 protein concentration was elevated compared to non-infarcted regions, confirming the PCR results (**B**). The ratio of phosphorylated YAP1 to non-phosphorylated YAP1 declined in the infarcted region of the heart compared to the non-infarcted region (**C**). Western blot image shows change in concentration of phosphorylated and non-phosphorylated YAP1 (**D**). *n* = 3, * *p* < 0.05, *** *p* < 0.001, **** *p* < 0.0001. Fold changes are shown as the mean ± SEM. All samples were run in triplicate.

**Figure 6:**
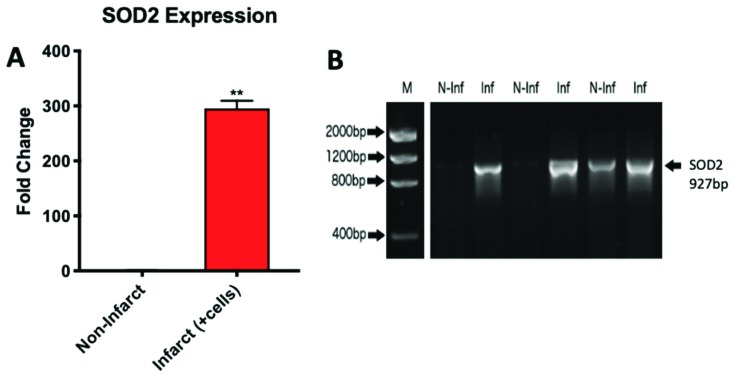
SOD2 transcripts are elevated in the infarcted zones of the sheep heart. The expression of this gene, downstream in the Hippo pathway, was significantly elevated in the infarcted repair region of the sheep heart indicating a shutdown of the inhibitory pathway (**A**). The size of the SOD2 transcript amplified by PCR was confirmed via gel imaging (**B**). *n* = 3, ** *p* < 0.01. Fold changes are shown as the mean ± SEM. All samples were run in triplicate.

The increasing evidence for a role of the Hippo pathway in cardiac regeneration in small animal models led us to investigate YAP1 in a sheep model of myocardial infarction and repair [40,41,42]. In this model, carboxyfluorescein succinimidyl ester (CFSE) labeled neonatal sheep CPCs expressing islet-1 and early-stage markers were administered by intramyocardial injection into infarcted sheep hearts to promote cardiovascular repair (Figure 4). These cardiovascular progenitor cells isolated from sheep were previously reported by our group to be comparable to the islet-1+ human CPCs studied routinely in our laboratory [43]. Control infarcted sheep hearts that were not injected with CPCs showed no change in YAP1 expression when comparing the infarcted region and the non-infarcted region of the heart. The infarcted zones of the cell-injected sheep hearts, however, had significantly higher YAP1 expression levels compared to their respective non-infarcted regions as observed by RT-PCR analysis (Figure 5A): YAP1 PCR infarct without cell treatment (0.993 ± 0.047-fold change), YAP1 PCR infarct with cell treatment (2.554 ± 0.049-fold change, *p* = 0.001). YAP1 protein levels were found to be elevated in infarct zones of cell injected sheep hearts compared to their respective non-infarcted regions (Figure 5B): YAP1 protein infarct with cell treatment (2.912 ± 0.355-fold change, *p* < 0.0001). As an indicator of Hippo pathway inhibition, we measured changes in phosphorylated-YAP1 (P-YAP1) levels in the infarcted and non-infarcted zones of the cell-injected sheep heart (Figure 5C,D): Phosphorylated YAP1 infarct with cell treatment (0.7046 ± 0.055-fold change, *p* < 0.05). Damaged cardiac tissue had lower concentrations of P-YAP1, as well as higher levels of free, non-phosphorylated YAP1. In addition, transcripts encoding SOD2, a downstream target of YAP1, were significantly elevated in the infarcted regions of cell-injected sheep hearts relative to the non-infarcted regions (Figure 6): SOD2 expression in infarct zone of cell treated sheep (295.4 ± 14.14-fold change, *p* < 0.01). SOD2 activation has been reported to be indicative of cell survival and activation of a protective, reparative mechanism [44,45]. The results demonstrate that YAP1 expression levels, which are known to be protective, are further elevated in the zone of repair when neonatal YAP1-expressing CPCs are initially introduced post-infarction in large animals. In a parallel model, transgenic YAP1 overexpressing adult mice have significantly improved cardiac repair to an extent comparable to that of neonates [46]. In sheep, pretreatment of adult CPCs with microgravity may therefore improve the outcome of cardiac repair.

### 2.5. Adult CPCs Treated with 1uM of 17-AAG Demonstrated Increased Levels of YAP1 and SOD2

**Figure 7:**
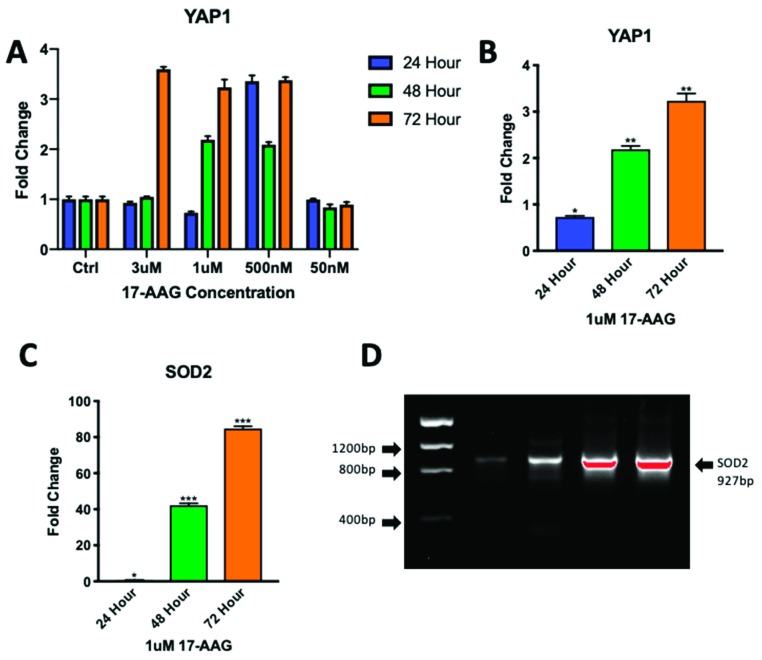
Adult CPCs treated with 17-AAG demonstrate upregulated YAP1 and SOD2 expression. Adult CPCs were treated with four concentrations of drug over three different time points for treatment optimization (**A**). 1 uM of drug produced the most consistent results with YAP1 expression increasing after 72 h (**B**). SOD2 expression was elevated in a similar pattern with higher fold changes (**C**). Gel electrophoresis was used to confirm the size of the SOD2 transcript (**D**). From left: Ladder, non-treated control, 24, 48, and 72 h treatment of 1uM 17-AAG. * *p* < 0.05, ** *p* < 0.01, *** *p* < 0.001. Fold changes are shown as the mean ± SEM. All samples were run in triplicate.

In order to achieve regenerative capacity comparable to that of neonatal CPCs in adults, the adult CPC transcriptome would need to be modified to induce expression of genes such as YAP1 that promote repair. We have shown that spaceflight is capable of elevating YAP1 in adult CPCs and that these effects can be simulated on earth by the use of a clinostat; however, the availability of such a device is very limited. Alternative methods of conditioning adult CPCs to express higher levels of YAP1 would be beneficial. Heat shock protein 90 (Hsp90) is a known regulator of the Hippo pathway that acts via the functional induction of LATS1 and LATS2 activity, the immediate upstream phosphorylators of YAP1. Using an Hsp90 inhibitor, 17-allylamino-17-demethoxygeldanamycin (17-AAG), we were able to induce adult CPCs to express higher levels of active, non-phosphorylated YAP1. At a concentration of 1uM of 17-AAG, YAP1 expression was elevated starting at 48 h (Figure 7A,B): 48 h 1uM 17-AAG YAP1 (2.19 ± 0.073-fold change, *p* < 0.01), 72 h 1uM 17-AAG YAP1 (3.24 ± 0.157-fold change *p* < 0.01).

In addition to YAP1 expression, we examined transcripts encoding a downstream target of the Hippo signaling pathway, SOD2, which has a protective effect on cells. SOD2 was significantly elevated in expression after 48 h of treatment with 1 uM 17-AAG following the same trend as YAP1 expression (Figure 7C,D): 48 h 1 uM 17-AAG SOD2 (42.2 ± 1.07-fold change, *p* < 0.001), 72 h 1 uM 17-AAG (84.76 ± 1.32-fold change *p* < 0.001).

### 2.6. miRNA-302a is Elevated in Adult CPCs Treated with 17-AAG

**Figure 8:**
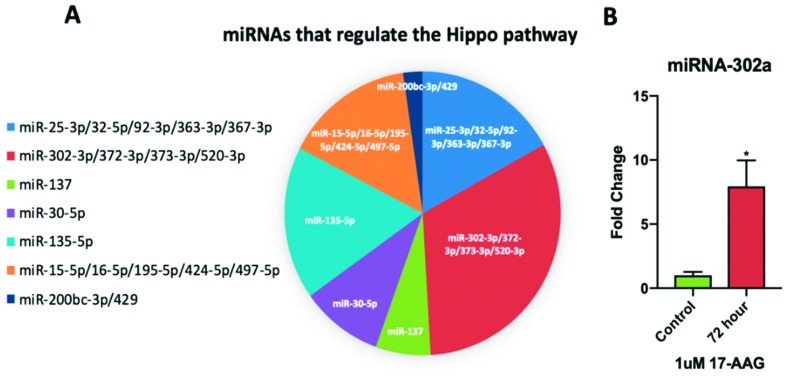
The miRNA-302 cluster is a significant regulator of the Hippo pathway (**A**). Adult CPCs treated with 1uM of 17-AAG for 72 h had elevated miRNA-302a transcript levels compared to non-treated control cells (**B**). * *p* < 0.05. Fold changes are shown as the mean ± SEM. All samples were run in triplicate.

Elevated miRNA-302 expression has been shown to induce cardiac regeneration in mice [47]. miRNA-302a is part of the miRNA-302 cluster that regulates the Hippo pathway (Figure 8A). Compared to non-treated adult CPCs, the cells treated with 1uM 17-AAG for 72 h demonstrated elevated levels of miRNA-302a (Figure 8B): 72 h 1uM 17-AAG miRNA-302a (7.94 ± 2.03-fold change, *p* < 0.05). The induction of miRNA-302a after 72 h of treatment corresponds with the YAP1 and SOD2 induction patterns shown above.

## 3. Discussion

The response of adult CPCs to reduced gravitational force has been studied under both real and simulated microgravity conditions in a series of experiments that were previously performed by our laboratory [4,13,17]; however, Hippo/YAP signaling was not extensively addressed. In the present study, we focused on the effect of real and simulated microgravity on the Hippo signaling pathway in adult CPCs, as well as the potential implications of these findings in cell-based repair. Under normal conditions, the Hippo signaling pathway is active in adults, and consequently, YAP1 expression is minimal. Here, we provide evidence that microgravity inhibits the Hippo pathway, driving adult cardiac cells to express higher levels of active YAP1.

We have reported that adult CPCs have elevated YAP1 transcript levels after 12 days of culture aboard the ISS and upon returning to normal gravity, are viable and proliferate actively after microgravity exposure [4]. Upon further investigation, we find that after 30 days of culture aboard the ISS, YAP1 induction is reduced compared to the 12-day flight sample. These findings demonstrate that microgravity has short term effects on YAP1 induction in adult CPCs. Temporary YAP1 activation dedifferentiates mature hepatocytes into progenitor cells that are more proliferative and capable of regeneration [48]. Transient induction of YAP1 transcripts in adult cardiac cells could prove to be beneficial based on the role of YAP1 in organ growth and proliferation, whereas permanent over-expression of YAP1 may result in oncogenic behavior [20,21,49,50,51]. It is important to note that the effects of microgravity following spaceflight are temporary in our cells, as well as in the hearts of astronauts. Interestingly, upon returning from space expeditions, astronauts temporarily present with enlarged or abnormally shaped hearts [52]. Considering the Hippo pathway’s role in regulating organ size, it is plausible to suggest that this signaling pathway may be inhibited not only in cardiovascular stem cells but in the heart itself as a consequence of spaceflight.

Simulated microgravity experiments performed in our lab demonstrate similar changes in YAP1 transcripts compared to cells flown in space. The application of these findings in a simulated microgravity setting are significant because the results are achievable in the absence of space radiation which has been shown to cause DNA damage and has negative effects on the cardiovascular system, such as fibrosis and increased atherosclerosis [53,54,55]. The use of simulated microgravity provides the beneficial effects of spaceflight without the increase in radiation exposure, making it a potentially valuable tool for microgravity-related applications on Earth. Activation of YAP1 induces expression of SOD2, a marker of cell survival that functions in the mitochondria to clear toxic reactive oxygen species, preventing apoptosis [56]. The dual upregulation of both YAP1 and SOD2 in adult CPCs is indicative of beneficial cellular modifications that lead to a more proliferative cell which migrates more efficiently and is more inclined to survive. We have previously shown that microgravity activation does not impair differentiation in adult CPCs [4].

miRNA-302a, which activates the miRNA cluster 302/367 consisting of miR-302a-d, miR-367 and a number of other miRNAs, is a significant regulator of Hippo pathway activity [57]. In mice, it has been shown that miRNA-302a is directly responsible for inhibiting the activity of LATS2, making it a key target for inducing YAP1 expression [58]. Similarly, repression of LATS2 activity via 17-AAG treatment elevated YAP1 transcript levels. We have demonstrated that this effect was mediated in part by an induction of miRNA-302a which has been shown to promote a more proliferative cell state [59] with the ability to reintroduce the regenerative effects commonly found in neonates [60].

The age-dependent response to cardiac injury that distinguishes adults and neonates is due, in part, to differing gene expression profiles. Neonatal CPCs are prime candidates for cardiovascular transplant studies due to their enhanced regenerative abilities and proliferative nature [60,61]. YAP1 is a gene that is highly expressed in neonates due to its role in cardiac development; however, in adults, YAP1 expression is limited [62]. In the context of cardiac repair, YAP1 plays a large role in cell survival and proliferation [46,63,64]. YAP1 overexpression in adult mice is sufficient to reintroduce regenerative potential normally found in neonates [27,46]. The ability to reintroduce YAP1 expression in adult CPCs may therefore increase the efficacy of regeneration in these cells. Verification of these findings in large animal models, which have not yet been investigated, would provide support for this concept in a model that is more clinically relevant for humans [37,65,66]. Sheep have a similar heart shape, resting heart rate, and blood pressure as humans and can provide valuable information regarding safety for cell-based repair [67]. We show that neonatal CPCs expressing high levels of YAP1 are retained for prolonged periods in our sheep model. Whether or not YAP1-induced adult CPCs will have comparable regenerative abilities remains to be determined. Future studies are needed to investigate how YAP1 overexpressing adult cells will behave following transplantation.

Our demonstration that 17-AAG mediates YAP1 induction in adult cells, accompanied by elevated SOD2 expression and miRNA-302a transcript levels, may therefore prove to be a valuable alternative preconditioning treatment. 17-AAG is a derivative of geldanamycin, a toxic Hsp90 inhibitor, and has been a focus in cancer research due to its availability and reduced toxicity [68]. The FDA has previously approved 17-AAG for clinical use and has placed it on a list of approved small molecule inhibitors, making it a safe alternative to induce YAP1 in humans.

We conclude from this study that microgravity activates YAP1 expression in adult cardiovascular progenitor cells. These findings have potential benefit for cardiovascular repair. Our data suggests that further studies defining the functional and safety implications of microgravity-activated cells in a sheep model of cardiovascular repair would provide valuable insight regarding microgravity-mediated conditioning in vivo. Inducing adult CPCs to over-express YAP1 is a first step towards conditioning these progenitors to have reparative potential closer to that of neonatal cells. Additional effects on the transcriptome still need to be defined, but as we continue to explore the outskirts of space, understanding the biological effects of microgravity on human cells and organs will allow us to adapt the human body to these effects in space and/or potentially apply this information in a beneficial way on Earth.

## 4. Materials and Methods

### 4.1. Isolation of Cardiac Progenitor Cells

Human and sheep cardiac progenitor cells were isolated as previously described by Fuentes et al. [30] and Hou et al. [43], respectively. The Institutional Review Board at Loma Linda University approved the protocol for using discarded human cardiovascular tissue obtained after cardiac surgery without the use of identifiable patient information, following a waiver of informed consent. All animal studies were approved by the Institutional Animal Care and Use Committee of Loma Linda University and performed within the regulations of the Animal Welfare Act (IACUC approval #:8160018, 27 May 2016). Sheep cells were harvested from right atrium tissue. Tissue samples were digested and cells were re-suspended. Cells were screened for the presence of markers of early state progenitor cells and isolated based on their expression of markers such as SSEA-4, Mesp1, and the co-expression of high levels of islet-1 and low levels of c-Kit.

### 4.2. Simulated Microgravity

CPCs were cultured in Biocell hardware, depicted by Lu et al. [69], on a two-dimensional clinostat (Bioserve, Boulder, CO, USA) as previously described by Fuentes et al. [13]. In the present study, CPCs were seeded in a total of 7 Biocells, growth area of 5.5cm, and exposed to clinorotation which involves constant rotation perpendicular to the force of gravity. The average centrifugal force on the cells along the edge of the Biocell is <0.5 mG. For the 72 h and 7-day simulated microgravity studies, Biocells were seeded with 2 × 10^5^ cells and 1 × 10^5^, respectively. The rotation rate of the Biocells was held constant with an average speed of 3.94 ± 0.01 rotations per minute. During clinorotation, cells were gassed with 5% CO2 and 95% oxygen. Control cells were cultured under static conditions at similar oxygen content for matched lengths of time. Upon reaching confluency, cells were trypsinized, counted, and stored in Trizol for RNA purification at a later time. Viability was ≥98% at recovery as determined by trypan blue assay. The design was intended to facilitate identification of the growth and seeding parameters in the hardware planned for flight (Biocells) as well as to provide information regarding the biological effects of simulated microgravity on the cardiovascular progenitor cells.

### 4.3. Culture of CPCs Aboard the ISS

Adult and neonatal CPCs were cultured in space aboard the ISS in 16 Biocells (8 for each group) as previously described by Baio et al. [4]. Adult CPCs were seeded into Biocells at two different cell densities, 5000 and 7500, and launched aboard SpaceX CRS-11. Upon arrival to the ISS, growth media was immediately replaced, and cells were fed on a regular schedule thereafter. On the twelfth day of culture aboard the ISS, the Biocells initially seeded with 7500 cells were fixed in RNAProtect (Qiagen, Valencia, CA, USA) and stored at −80. On the thirtieth day, the remaining cells (814,400 cells, 93% viability) were returned to Earth live, retrieved, and either fixed in RNAProtect immediately or used for functional assays [4].

### 4.4. Sheep Model of Myocardial Infarction

Sheep (<1 year old) were infarcted via left anterior descending coronary artery ligation. Three to four weeks following infarction, sheep were injected with 10 million sheep neonatal cardiac progenitor cells. Two months after cell injection, the sheep were euthanized and their hearts were collected. Hearts were mapped and sections were taken according to their proximity to the infarct site. RNA and protein were isolated from frozen tissue. Six-micron frozen sections of sheep cardiac tissue were cut for documentation of CFSE (Biolegend, San Diego, CA, USA) labeled cell retention and for immunostaining. Labeling for endothelial cells expressing von Willebrand Factor (vWF), was performed using anti-vWF antibody product number A0082, lot 20011542, at a 1:200 dilution (DAKO, Carpinteria, CA, USA). The secondary antibody used was conjugated with Alexa Fluor 647 product number ab150075, lot GR114884-1 (Abcam, Cambridge, MA, USA) at a 1:200 dilution.

### 4.5. Flow Cytometry

Cells were labeled using antibody concentrations provided by the manufacturer. Fluorescence was analyzed using a MACSquant analyzer (Miltenyi Biotec, Auburn, CA, USA) and quantified via FlowJo software (Ashland, OR, USA). Gating was uniformly applied to all samples. Antibodies used for flow cytometry experiments are listed in Table 1.

### 4.6. Treatment of Adult Cardiac Progenitor Cells with 17-AAG

Adult CPCs were cultured at a density of 35,000 cells per well in a 6-well plate (Genesee Scientific, San Diego, CA, USA) and allowed 24 h to adhere. Cells were then treated with 17-AAG (Minneapolis, MN, USA) infused growth media, dimethylsulfoxide (DMSO) infused growth media, or control growth media. Drug concentrations were from a working stock of 100 mM and diluted with DMSO. Each working stock was made so that the final volume of drug-infused media for each concentration contained the same amount of DMSO. Cells were treated for 24, 48, and 72 h with 0.05–3 uM of drug in order to optimize treatment conditions for increased YAP1 expression.

### 4.7. Western Blot and Protein Purification

Tissue samples were dissected into 1–5 mg sections for chemical digestion. Samples were held at constant agitation for 2 h at 4 °C in protein lysis buffer consisting of RIPA buffer, 0.5M EDTA, protease inhibitor cocktail, sodium orthovanadate, and sodium fluoride, before being centrifuged at 14,000× *g*, and aliquoted for use. Protein concentrations were quantified using the Micro BCA Protein Assay Kit (ThermoFisher, Waltham, MA, USA). An automated, gel-free western blotting system was used following the protocol provided by the manufacturer (ProteinSimple Wes, San Jose, CA, USA) to quantify protein levels. Antibodies used are shown in Table 2 below (Cell Signaling, Danvers, MA, USA).

### 4.8. RNA Purification and RT-PCR

Purification of total RNA from sheep samples was performed using the miRNeasy Mini Kit (Qiagen, Valencia, CA, USA) following the manufacturer’s instructions. In short, 50 mg of sample was homogenized in Qiazol (Qiagen) before being centrifuged through the provided RNeasy Mini spin column followed by a series of washes with provided buffers. RNA was eluted with 40 µL RNase-free water. cDNA was synthesized with Superscript III (Invitrogen, Carlsbad, CA). PCR plates were run under the following conditions: 94 °C for 10 min, 94 °C for 15 s, 58 °C for 1 min, 72 °C for 30 s, repeated for 45 cycles. Primers for our genes of interest were created using NCBI Primer-BLAST and are displayed in Table 3. RT-PCR was performed with a Bio-Rad CFX96 Touch Real-Time PCR Detection System (Bio-Rad, Hercules, CA, USA).

## Figures and Tables

**Table 1:** Antibodies Used for Flow Cytometry.

Antibody	Manufacturer	Isotype	Species	Clone	Catalog No.	Lot No.
Islet-1	Abcam	IgG1	Mouse	1H9	ab86472	GR273015-3
cKit-Dylight650	Novus Biologicals	IgG2B Kappa	Mouse	2B8	NB100-77477c	B147020-A
CD56	Biolegend	IgG1	Mouse	5.1H11	362545	B245476

**Table 2:** Antibodies Used for Western Blotting.

Antibody	Manufacturer	Sample Used (mg/mL)	Antibody Dilution	Species	Size (kDa)	Catalog No.	Lot No.
GAPDH	Cell Signaling	0.4	1:50	Mouse	37	97166S	4
YAP1	Cell Signaling	0.4	1:200	Rabbit	65–75	14074S	2
Phosphorylated YAP1	Cell Signaling	0.4	1:10	Rabbit	65–75	13008S	5

**Table 3:** Primer Pairs Used for RT-PCR (5′ to 3′).

*Gene*	Forward Sequence	Reverse Sequence
GAPDH	TGCACCACCAACTGCTTAGC	GGCATGGACTGTGGTCATGAG
YAP1	TCCCAGATGAACGTCACAGC	TCATGGCAAAACGAGGGTCA
SOD2	ACCACGCGGCCTACGTGAAC	AGAAAGCCGAGTGTTTCCCTT
Sheep GAPDH	CCAGCCGCATCCCTGAGACAA	GACCCTCTTGGCGCCACCCT
Sheep YAP1	GCACCTTCGACAGTCTTCCT	TTCTCTGGTTCATGGCAAAACG

## Abbreviations

ISS	International Space Station
CPC	Cardiac progenitor cell
YAP1	Yes-associated protein 1
LATS1/2	Large tumor suppressor 1/2
SOD2	Superoxide dismutase 2
17-AAG	17-allylamino-17-demethoxygeldanamycin
DMSO	Dimethylsulfoxide
CFSE	Carboxyfluorescein succinimidyl ester
